# The basic research factors questionnaire for studying early childhood caries

**DOI:** 10.1186/s12903-017-0374-5

**Published:** 2017-05-19

**Authors:** Judith Albino, Tamanna Tiwari, Stuart A. Gansky, Michelle M. Henshaw, Judith C. Barker, Angela G. Brega, Steven E. Gregorich, Brenda Heaton, Terrence S. Batliner, Belinda Borrelli, Paul Geltman, Nancy R. Kressin, Jane A. Weintraub, Tracy L. Finlayson, Raul I. Garcia, Early Childhood Caries Collaborating Centers

**Affiliations:** 1 Center for Native Oral Health Research (CNOHR), University of Colorado Anschutz Medical Campus, Aurora, CO USA; 2 Department of Applied Dentistry, School of Dental Medicine, University of Colorado Anschutz Medical Campus, Aurora, CO USA; 3 Center to Address Disparities in Children’s Oral Health (CAN DO), School of Dentistry, University of California San Francisco, San Francisco, CA USA; 4 Center for Research to Evaluate and Eliminate Dental Disparities (CREEDD), Boston University Henry M. Goldman School of Dental Medicine, Boston, MA USA; 5 Department of Medicine, School of Medicine, University of California San Francisco, San Francisco, CA USA; 6 Section of General Internal medicine, Department of Medicine, Boston University School of Medicine, Boston, MA USA; 7 VA Boston Healthcare System, Jamaica Plain, MA USA; 8 University of North Carolina School of Dentistry, Chapel Hill, NC USA; 9 Graduate School of Public Health, San Diego State University, San Diego, USA

**Keywords:** Dental caries, Children, Surveys, Risk factors, Oral health, Parent/caregivers, Common data elements

## Abstract

**Background:**

We describe development of the Early Childhood Caries (ECC) Basic Research Factors Questionnaire (BRFQ), a battery of measures assessing common potential predictors, mediators, and moderators of ECC. Individual-, family-, and community-level factors that are linked to oral health outcomes across at-risk populations are included. Developing standard measures of factors implicated in ECC has the potential to enhance our ability to understand mechanisms underlying successful prevention and to develop more effective interventions.

**Methods:**

The Early Childhood Caries Collaborating Centers (EC4), funded by National Institute of Dental and Craniofacial Research, developed the BRFQ, which was used across four randomized trials to develop and test interventions for reducing ECC in at-risk populations. Forty-five investigators from across the centers and NIDCR were involved in the development process. Eight “measures working groups” identified relevant constructs and effective measurement approaches, which were then categorized as “essential” or “optional” common data elements (CDEs) for the EC4 projects.

**Results:**

Essential CDEs include 88 items, with an additional 177 measures categorized as optional CDEs. Essential CDEs fell under the following domains: oral health knowledge, oral health behavior, utilization/insurance and cost, parent/caregiver dental self-efficacy, quality of life, caregiver and family characteristics, and child characteristics.

**Conclusions:**

The BRFQ makes available a battery of measures that support efforts to understand population risk factors for ECC and to compare oral health outcomes across populations at risk. The BRFQ development process may be useful to other clinical research networks and consortia developing CDEs in other health research fields.

**Trial registration:**

All the trial that used the BRFQ were registered at Clinicaltrial.gov NCT01116726, April 29, 2010; NCT01116739, May 3, 2010; NCT01129440, May 21, 2010; and NCT01205971, September 19, 2010.

## Background

Significant disparities are seen in the oral health of children across racial and ethnic minority groups, including Latino, American Indian and Alaska Native, and African American populations in the United States. Investigators conducting research with these populations have assessed child and family socio-demographic, biological, behavioral, social, and psychological variables that are theorized to influence oral health. Building on the results of this work has been challenging because of varying methods employed in defining and measuring these constructs. The need to identify predictors, mediators and moderators that influence intervention effects and compare them across populations requires consistency in measurement. The National Institutes of Health and other institutions have emphasized the need to develop common data elements (CDEs) to facilitate data interoperability in large clinical research networks and consortia [1]. Developing a standardized approach to measuring key characteristics of communities, families, and individuals with respect to preventing oral disease in children should facilitate development and evaluation of interventions to enhance children’s oral health outcomes across a variety of communities.

The Early Childhood Caries Collaborating Centers (EC4) and their common Data Coordinating Center (DCC) are funded by the National Institute of Dental and Craniofacial Reseach (NIDCR). The Center to Address Disparities in Children’s Oral Health at the University of California San Francisco (UCSF), the Center for Research to Evaluate and Eliminate Dental Disparities at Boston University (BU), and the Center for Native Oral Health Research at the University of Colorado Denver (UCD) were funded to conduct research that would add to knowledge regarding means of reducing oral health disparities through ECC prevention in at-risk communities. NIDCR required these Centers to develop essential CDEs, including identification of constructs reflecting important characteristics of children and their parents/caregivers. Clinical trials conducted by all Centers collected CDEs in a standardized fashion to facilitate comparisons of risks and outcomes across the studies.

The Basic Research Factors Questionnaire (BRFQ) has been used in four EC4 clinical trials. It also has been used in observational studies to understand the risk factors for ECC within a given population and to compare risk factors across population groups. It was administered to parents/caregivers of young children aged 0–5 years. It has been used with low-income and underserved families, including low income Spanish and English-speaking Latinos in San Diego and Monterey Counties in California; with low-income, racially and ethnically diverse, underserved families living in urban, public housing developments in Boston; and with American Indian families living in reservation communities in the Southwest and the Northern Plains [2].

This paper describes the process of developing the BRFQ, providing ECC prevention investigators with an opportunity to more knowledgeably choose all or some of the battery for use in their research. Doing so will facilitate comparability with other available data related to the efficacy of oral disease prevention efforts in diverse populations. In turn, this more standardized approach to defining and measuring variables will assist the larger scientific community in making supportable generalizations across studies and lead to more confident understanding of the probable outcomes of various prevention approaches and strategies.

## Methods

As work on clinical trials within their respective Centers began, NIDCR convened EC4 investigators to develop a common, parent-report instrument that assessed factors potentially related to ECC that became the BRFQ. The BRFQ was to be the vehicle for collecting information on variables likely to be predictors, mediators, or moderators of pediatric oral health. Using the conceptual framework described by Fisher-Owens et al. [3] as a starting point, we built a comprehensive battery of measures that were expected to be associated with early childhood caries (ECC) and responses to prevention interventions at three levels:

1. individual characteristics of children and their parents/caregivers, including sociodemographics, oral hygiene practices, dental care utilization, and pediatric oral health-related quality of life;
2. parent/caregiver level characterstics, including oral health knowledge, attitudes and behaviors, and psychosocial factors such as chronic stress, social support, and self-reported parental oral health.
3. community-level influences, including social environment, physical environment, and community cultural components.

### Measure selecting criteria

The EC4 followed a systematic multi-step process in selecting and defining measures included in the BRFQ. Several of the measures were previously validated at the time of selection, and other measures are undergoing the process of validation.

1. Development of the Measures working groups: Forty-five investigators from across the Centers and NIDCR were involved in the process, functioning as eight measures working groups, each focused on one or more categories of constructs and their measurement (Table 1). Measures working groups generally included at least one member from each Center. All measures working groups initially met face-to-face at a kick-off meeting of the Centers in San Francisco. Some groups subsequently invited participation from colleagues outside the Centers when their expertise was needed. Each group met on 9 to 12 occasions via conference calls.

**Table 1:** Desciption of Measure Working Subgroup Members

Working group	Chair	Members	Disciplines
Behavioral Intervention	Paul Spicer (UCD, now at University of Oklahoma-UOk)	Belinda Borrelli (now BU), Barbara Heckman (UCSF), Karen Fehringer (UCD), Margaret Walsh (UCSF), Melissa Riddle (NIDCR), Michelle Henshaw (BU), Nancy Kressin (BU), Ruth Nowjack-Raymer (NIDCR), and Tracy Finlayson (San Diego State University-SDSU).	anthropology, behavioral science, dental hygiene, dentistry, educational research, epidemiology, health services research, pediatric dentistry, public health
Sociodemographic	Stuart Gansky (UCSF)	Clemencia Vargas (University of Maryland-UMd), Jan Beals (UCD), Jane Weintraub (UCSF, now at University of North Carolina-UNC)	biostatistics, dentistry, demography, public health, social psychology
Health Service Utilization/Insurance	Tracy Finlayson (SDSU)	Cindy Cadoret (BU), Daniel Brooks (BU), Steve Silverstein (UCSF), Terry Batliner (UCD)	dental hygiene, dentistry, epidemiology, health service research, public health, social psychology
Oral Health Knowledge and Behavior	Judith Albino (UCD)	Angela Brega (UCD), Clemencia Vargas (UMd), Kristin Hoeft (UCSF), and Margaret Walsh (UCSF) and Jane Weintraub (UNC).	dentistry, dental hygiene, health education, medical sociology, public health, social psychology
Health Status, Health History, and Development	Gloria Mejia (UCSF, now at University of South Australia)	Karen Fehringer (UCD), Paul Geltman (BU), Rosalia Mendoza (UCSF, now at Kaiser Permanente)	dentistry, epidemiology, pediatrics, public health
Community Level-Social Environment, Culture	Paul Spicer (UCD, now at UOk)	Judith Barker (UCSF), Kristin Hoeft (UCSF), Maria Rosa Watson (private consultant)	anthropology, health education, pediatric dentistry, public health
Psychosocial Factors	Angela Brega (UCD)	Judith Albino (UCD), Nancy Kressin (BU), Steve Gregorich (UCSF), Tracy Finlayson (SDSU)	biostatistics,, epidemiology medical sociology, social psychology, public health
Cost Measures	Joan O’Connell (UCD)	Brenda Heaton (BU), Jane Weintraub (UCSF, now at UNC), Margaret Walsh (UCSF), Ruth Nowjack-Raymer (NIDCR), Sally Stearns (UNC), and Susan Griffin (Centers for Disease Control and Prevention).	educational research, epidemiology, dental hygiene, general dentistry, health economics.
2. Based on their prior research experience and literature reviews, groups identified critical constructs to be measured across all studies in the three Centers, located and evaluated validated measures of such constructs, and, when necessary, developed new measures. The groups then developed recommendations regarding which constructs and measures should be utilized for the EC4 clinical trials. Each project evaluated, at the construct level, the compiled measures that the work groups proposed. Issues of respondent burden, relative value of different response options, literacy level, translation and cultural applicability, and areas of construct overlap were discussed. Next, recommended measures were reviewed by investigators across the three Centers and were revised with input from investigators experienced in using the measures in diverse communities.
3. In next step, the DCC compiled items, created consistent formatting, and facilitated other administrative and data management streamlining. Measures working groups reviewed and assisted with developing consistent administration instructions and response options to be used across projects. The BRFQ was developed for use both as an interviewer-administered and as a self-administered instrument.
4. Cultural appropriateness: Throughout the measures development process, the measures working groups ensured that items were culturally appropriate and written at no higher than a 6^th^ grade reading level. Each Center had advisory boards and advisory committees that had members and stakeholders form the participating communities. The measures developed by the working groups were reviewed by these members for cultural appropriateness.
5. Role of EC4 Steering Committee: The eight measures working groups reported their recommendations in their respective domains to the EC4 Steering Committee (Fig. 1). The EC4 Steering Committee comprised the EC4 Center directors and associate directors, the DCC director, and the NIDCR Program Official as primary members. The EC4 Steering Committee provided oversight of the eight measures working groups and developed procedures, such as for identifying “essential” (Table 2) and “optional” CDEs (Table 3). Each Center used several items that were specific to the population with which the Center worked, and these variables were considered optional for the other Centers. Categorization of variables as optional was based on concerns related to respondent burden, putative mechanisms of action (e.g., investigators conducting Motivational Interviewing interventions wanted to measure motivation), relative value in terms of established validity and reliability of available measurement scales, cultural applicability, and areas of overlapping constructs.

**Fig. 1:**
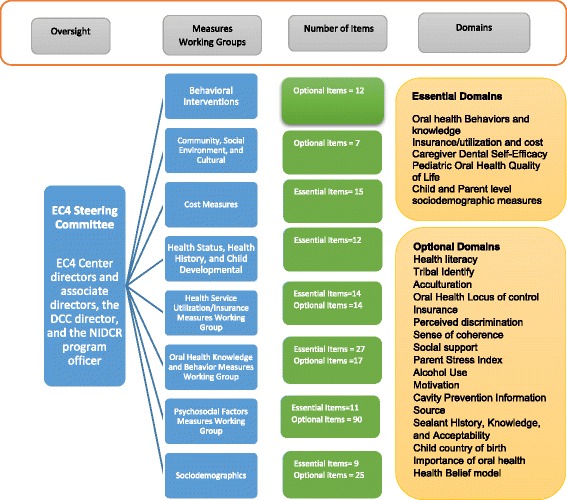
Overview of the BRFQ development process

**Table 2:** Explanation of the essential CDEs shared by all three ECC centers

Domain (number of items)	Description	Scoring	Levels
Parental Oral Health Behaviors (12)	Items related to parental oral health behavior assess oral hygiene routines and feeding practices. The overall behavior score represents the percentage of oral health behavior items that were answered with an “adherent” response. Adherent means the participant reported following the recommended oral health behavior.	0-100%	Parent/Family
Insurance/Utilization & Cost (29)	These items assess access to dental care and utilization of dental health care for prevention and treatment for dental caries.	N/A	Child/Parent/Family
Parental Oral Health Knowledge (15)	This measure assesses parental knowledge related to oral hygiene routines and feeding practices. The overall knowledge score represents the percentage of oral health knowledge items answered correctly.	0–100%	Parent/Family
Parent/Caregiver Dental Self-Efficacy (11)	The overall self-efficacy score represents how sure participants are that they can engage in recommended behavior to take care of their children’s teeth. The overall self-efficacy score is the mean of responses to all items.	1–5 1 = Strongly disagree 5 = Strongly agree	Parent/Family
Pediatric Oral Health Quality of Life (12)	This measure assesses the extent to which a child’s functioning is affected by negative oral health experiences.	1–4 1 = all the time 4 = Did not happen	Child
Parent/Caregiver and Family Sociodemographics (6)	Age, education, employment status, income, parent/caregiver relationship to the child and number of household members	N/A	Parent/Family
Child Sociodemographics (3)	Race, ethnicity, and age	N/A	Child

**Table 3:** Explanation of optional CDEs by specific centers

Domains (number of items)	Center using the variable	Description (number of items)	Range	Levels
Health Literacy (1)	BU and UCSF	Single Item Literacy Screener (SILS) of Chew et al. (2004) and Morris et al. (2006)	1–5 1 = always 5 = never	Parent/Caregiver
Health Literacy (2)	UCD	3 items adapted from screening items developed by Chew et al. (2004) and Morris et al (2006)	1–5 1 = always 5 = never	Parent/Caregiver
Tribal Identity (2)	UCD	Tribal identity measures items assessing facility with tribal language and tribal values	1–5 1 = always 5 = never	Child
Acculturation (13)	BU and UCSF	The revised Acculturation Rating Scale for Mexican-Americans (ARSMA-II)	1–5 1 = Not at all 5 = All the time	Parent/Family
Oral Health Locus of Control (9) Subscales - Internal locus of control (3) External locus of control - powerful others (3) External locus of control - chance (3)	UCD	Attitudes about who or what has control over their child’s oral health outcomes (i.e., the parents themselves, the dentist, or chance)	1–5 1 = Strongly disagree 5 = Strongly agree	Parent/Family
Official Tribal Affiliation (3)	UCD	This measure includes items related to enrollment in the tribe	1 = yes 0 = no 2 = pending	Parent/Family
Insurance (7)	UCD	This measure included items related to Indian Health Services	1 = yes 0 = no	Parent/Family
Perceived Discrimination (9)	UCD	The perceived discrimination measure represents the amount of discrimination participants feel they are subject to, on account of being American Indian	1–4 1 = Never 4 = Often	Parent/Family
Sense of Coherence (13) Comprehensibility (5) Meaningfulness (4) Manageability (4)	UCD	Degree to which participants feel the world makes sense and has meaning	1–7 Higher numbers indicate stronger coherence	Parent/Family
Social Support (4)	UCD	Degree to which participants believe they have others available to help them when needed	0–1 0 = No available support 1 = Support available	Parent/Family
Chronic Stress (17 Subscales - Expectations (3) Location hassles (5) Community family dysfunction (2) Community risky behaviors (5) Community economic distress (2)	UCD	Ongoing stress related to personal expectations, hassles associated with the local community, and community dysfunction	1–4 1 = Strongly disagree 4 = Strongly agree	Parent/Family
Parent Stress Index (8)	UCSF	8 parent stress items adapted from Detroit Dental Health Project’s adaption of the short version of Abidin	1–5 1 = never 5 = almost always	Parent/Family
Alcohol Use (3)	UCD	A shortened version of the Alcohol Use Disorders Identification Test (AUDIT). Referred to as the AUDIT-C. The alcohol score provides an indication of the degree to which a participant drinks excessively.	0–12 Large numbers represent greater alcohol use	Parent/Family
Distress (6)	UCD	Amount of distress experienced in the last 30 days.	1–5 1 = none of the time 5 = all the time	Community Parent/Family
Motivation (12)	BU	Items are summed to indicate how motivated participants are to engage in a variety of behaviors concerning their child’s oral health	1–5 scale 1 = not at all 3 = somewhat want to 5 = very much	Parent/Family
Caregiver Stress (9)	BU	The measured represents caregiver stress using the Hassles Scale	1 = yes 0 = no	Parent/Family
Oral Health Knowledge (17)	BU and UCSF	The overall knowledge score represents the percentage of oral health knowledge items answered correctly.	Four items are true/false;13 items have these response options: Good for your child’s teeth, does not affect your child’s teeth, bad for your child’s teeth, don’t know, and prefer not to answer.	Parent/Family
Cavity Prevention Information Source (1)	UCSF	Where does the parent/caregiver get information about cavity prevention	TV, radio, dentist, family member, friends, magazine, doctor, WIC program	Parent/Family
Sealant History, Knowledge, and Acceptability (6)	UCSF	Past sealant history, knowledge and acceptability	1 = yes 0 = no Don’t know	Child
Child country of birth (3)	UCSF	This measure collects information about the country of birth	N/A	Child
Child birth order (1)	UCSF	Birth order	N/A	Child
Importance of engaging in behaviors to support child’s oral health (12)	BU and UCD	Items are summed to indicate participants’ perceived importance of engaging in a variety of behaviors concerning their child’s oral health.	1–5 scale 1 = not at all 3 = somewhat want to 5 = very much	Parent/Family
Physical Living Conditions (4)	BU and UCD	This measure assess the living conditions of the caregiver (having enough food, decent place to live, sufficient clothing and access to health care)	1 = yes 0 = no Don’t know and prefer not to answer are also options	Parent/Family
Health Belief Model (16) Subscales -Perceived susceptibility (3) Perceived severity (3) Perceived barriers (5) Perceived benefits (5)	UCSF, BU and UCD	The measure represents the parent/caregiver agreement level with items related to each domain.	1–5 1 = Strongly disagree 5 = Strongly agree	Parent/Family
6. Translations: Essential and optional CDEs were translated into Spanish and back translated to English to ensure proper translation by a single IRB-approved translation service. Following translation, bilingual team members from the BU and UCSF study teams reviewed the translations for dialects and idioms of local Spanish-speaking populations, and the BU and UCSF versions then were harmonized.
7. Pilot Testing: Pilot testing, including cognitive interviews after BRFQ administration, of both essential and study-specific optional items was conducted separately by each of the four trials as well as other projects in the three Centers, and final refinements were based on those results. If a trial planned to enroll Spanish-speaking participants, then the pilot testing included Spanish-language materials.

The measures working groups, their areas of focus, and major decisions and recommendations are detailed below.

### Measures working groups

1. Behavioral Interventions Measures Working Group –This group established a set of 11 oral health messages judged to be critical for ECC prevention (Table 4). These messages provided additional guidance for selecting CDEs – particularly those considered by the Oral Health Knowledge and Behaviors and the Psychosocial Factors Measures Working Groups. These messages were also incorportated into the interventions implemented by each Center, and measures were designed to capture content relevant to each message (e.g., oral health knowledge and behavior measures). The Behavioral Interventions Measures Working Group also developed a measure of perceived importance of oral health behaviors. These items mirrored the BRFQ self-efficacy items developed by the Psychosocial Measures Group (described below) and assessed parental motivation to engage in recommended parental oral health behaviors [4, 5]. Self-efficacy items ask “how sure” a participant is that he/she can engage in specific oral health-related behaviors, and the importance items assess participant perceptions of “how important” it is that they engage in those behaviors. Twelve optional items under the domain “Importance of engaging in behaviors to support child’s oral health” comprised the final set (Table 3).

**Table 4:** Behavioral Interventions Measures Working Group - Oral health Messages

1. How Tooth Decay Happens Cavities are caused by germs in the mouth.
2. Baby Teeth Are Important Because they do not stay in your child’s mouth very long, baby teeth are not that important.
3. Lift the Lip Parents checking their child’s teeth every month for changes or spots.
4. Take Your Child to the Dentist There’s no need to go to the dentist unless children have a problem with their teeth.
5. Protect Your Child’s Teeth With Fluoride It is best to use toothpaste with fluoride when brushing a child’s teeth. Getting fluoride varnish put on your child’s teeth.
6. Brush Daily How many times a day should a child’s teeth be brushed?
7. Limit Food and Drinks with Sugar How sure are you that you can: keep your child from eating frequent sweets (cake, candy)? How sure are you that you can: keep your child from drinking sugary drinks like soda, pop, or Kool-Aid?
8. No Bottles or Sippy Cups in Bed Good/Bad - Drinking milk from a sippy cup at bedtime.
10. Don’t Share Germs Using the same spoon to taste the food and feed the child. Sharing a toothbrush with your child.
11. Help Children Brush Up to Age Six At what age can a child brush his/her teeth by himself/herself?
2. Community, Social Environment, and Cultural Measures Working Group – This group began by identifying constructs at the community and social levels, for each Center and across Centers, that investigators thought could impact children’s oral health. They identified available measures and, when necessary, developed new measures. Among measures proposed by the group were water fluoridation, quality of housing, and perceptions of social environment (i.e., discrimination, violence, cohesiveness); the group recommended recording residential ZIP codes to enable linking to Census and Area Resource File data. However, community water fluoridation was not measured in the BRFQ because all participants in the CREEDD and CANDO trials lived in areas with community water fluoridation. Thus, investigators determined the more important issue for these randomized trials was asking about tap water consumption. Measuring community water fluoridation for CNOHR trials was difficult because the population was rural and had many soucres of water including well water. Also, water fluoridation was spotty and inconsistent in these areas. There were a total of seven optional CDEs under the domain “Physical Living Conditions” and “Alcohol Use” (Table 3).
3. Cost Measures Working Group – The goal of this group was to develop methodologies to uniformly measure societal and personal/familial costs of preventive interventions and also relative intervention costs in the context of possible treatment averted. The group collaborated with the Health Services Utilization/Insurance Group to develop a limited number of measures to address both utilization and costs. These measures address utilization of services for dental problems in dental clinics, hospital emergency departments, and hospital and outpatient surgery centers (where anesthesia often is used to treat severe ECC cases). Cost measures also address family costs related to travel for services and time spent at providers’ offices or clinics obtaining and waiting for services. The final set included 15 essential items under the domain “Insurance/Utilization & Cost” (Table 2).
4. Health Service Utilization/Insurance Measures Working Group – This group’s charge was to identify uniform questions related to dental service utilization. These included time since the last dental visit, age of first dental visit, reason for last visit, barriers to care, dental home status, types of services received, type of health insurance, delay in getting appointments, and perceptions related to the availability of services and child dental need. The group reviewed and selected items from a list that initially included multiple versions of questions related to access and utilization concepts at the individual, community, and state levels. The final set included a total of 14 essential items under the domains “Insurance/Utilization & Cost” (Table 2) and 14 optional items under the domains “Insurance”, “Cavity Prevention Information Source”, “Sealant History, Knowledge, and Acceptability” (Table 3).
5. Health Status, Health History, and Child Developmental Measures Working Group – This group identified measures of parent-reported oral and general health, quality of life, major medical conditions, medications, allergies, special needs and physical development of children. They identified some items from NHANES related to oral and general health status and selected the BU-developed Pediatric Oral Health-Related Quality of Life (POQL) measure [6]. They made suggestions for both the essential and optional lists in the areas of medications/allergies. The final set of recommendations included the 12 essential items under the domain “Pediatric Oral Health Quality of Life” (Table 2).
6. Oral Health Knowledge and Behavior Measures Working Group – This group focused on identifying existing measures to capture parent/caregiver oral health knowledge and behaviors. The group sought to identify or develop items that would capture parent/caregiver knowledge and behavior related to each of the 11 intervention targets specified by the Behavioral Intervention Measures Working Group (Table 1). They refined items to ensure age appropriateness for children who were to be enrolled and longitudinally assessed across all EC4 projects. The age range included birth to 6 years at the time of enrollment in the study and 3 to 8 years at longitudinal follow-up. The final set of recommendations included 27 essential items that were under the domains, “Oral Health Behaviors” and “Oral Health Knowledge” (Table 2) Optional items were under the “Oral Health Knowledge” domain and had 17 items (Table 3).
7. Psychosocial Factors Measures Working Group – Each Center had originally planned to assess psychosocial constructs, so the group began by exchanging their respective Centers’ proposed psychosocial measures and the rationales for these choices. The group then identified constructs of interest across Centers and began identifying and developing appropriate measures. The group eventually recommended measures related to several psychososcial constructs: perceived discrimination, social support, sense of coherence, chronic stress, parenting stress, psychological distress, self-efficacy, and constructs from the Health Belief Model [7–13]. Only the self-efficacy measure was included as an essential domain because the populations and interventions varied greatly across Centers and trials. The final set of recommendations included 11 essential items (Table 2) under the domain “Parent/Caregiver Dental Self-Efficacy”. Ninety optional items were under the domains “Perceived Discrimination”, “Social Support”, “Sense of Coherence”, “Chronic Stress”, “Parental Stress Index”, “Distress”, “Caregiver Stress”, “Oral Health Locus of Control”, and “Health Belief Model” (Table 3).
8. Sociodemographic Measures Working Group – The purpose of this group was to identify uniform questions related to relevant socio-demographic variables. Items were compiled from the National Cancer Institute’s Biomedical Informatics Grid, National Health and Nutrition Examination Survey [14] National Health Interview Survey [15] MacArthur Foundation Network on Socioeconomic Status and Health [16], and PubMed literature searches; these included items pertaining to children (age, sex, race, ethnicity, birth order, and country of birth), and to parents/caregivers (relationship to child, educational attainment, employment status, health literacy, language spoken, subjective social status, acculturation, tribal identity). Other family-level factors included number in household, household poverty status, residency longevity, food security, transportation access, and housing security. The final recommendation included nine essential items (Table 2) under the domains, “Parent/Caregiver and Family Socio-demographic” and Child Socio-demographic. Twenty-five optional items were under the domains, “Health Literacy”, Tribal Identity”, “Acculturation”, Official Tribal Affiliation”, Child county of Birth”, Child Birth Order” (Table 3).

For each clinical trial, the relevant university IRB reviewed and approved the BRFQ instrument protocol and the consent process.The UCD studies were approved by the Colorado Multiple Institutional Review Board. The UCSF study was approved by UCSF Committee on Human Research, the UCLA Medical Institutional Review Board and the San Diego State University Institutional Review Board. The BU study was approved by Boston University Medical Campus Institutional Review Board.

## Results

Selected measures were refined by the measures working groups and forwarded in March, 2009, to the EC4 Steering Committee for final review. Table 2 provides the final set of essential CDEs (88 items) and includes explanations and scoring criteria. A total of 7 domains were deemed to be essential: oral health knowledge (15 items), oral health behavior (12 items), utilization/insurance & cost (29 items), parent/caregiver dental self-efficacy (11 items), pediatric oral health quality of life (12 items), caregiver and family characteristics (6 items), and child characteristics (3 items). Table 3 provides a list of optional CDEs (177 items) and their definitions.

Across the four trials, baseline BRFQ data were collected for a total of 3,265 caregiver participants (Table 5). The method of administering the BRFQ varied across centers; UCD studies have used an Audio Computer-Assisted Self-Interview software system; the BU and UCSF studies used Computer Assisted Personal Interviews. Because of these differences and because the number of optional items used determined the length of each Center’s survey, the average time required to complete the baseline BRFQ also varied. The average time taken by UCD study participants to complete the BRFQ was 37 min. BU study participants needed 45–60 min and UCSF study participants completed the assessment in an average of 25 min.

**Table 5:** Demographics for All Three Centers

Centers	Sample size	Caregiver relation to the child (%)		Caregiver education (%)		Caregiver Poverty Status (%)		Caregiver race/ethnicity
UCD (Study I + Study II)	1603	Mother	84.0	<HS	24.9	Below FPL	78.6	American Indian (several different tribal affiliations)
		Father	10.4	HS or GED	32.5	Above FPL	21.4	
		Other	5.5	>HS	32.5			
				College grad+	10.1	Missing	0	
BU study	1036	Mother	94.7	<HS	22.3	Below FPL	88.9	White Hispanic, White Non-Hispanic, Black Hispanic, and Black Non-Hispanic
		Father	1.6	HS/GED	26.5	Above FPL	11.1	
		Other	3.7	>HS	22.3			
				College grad +	28.9	Missing	7.0	
UCSF study	597	Mother	88.1	<HS	37.8	Below FPL	74.8	Mostly White Hispanic, but also Asian, White Non-Hispanic, Black Hispanic, and Black Non-Hispanic
		Father	5.9	HS/GED	35.3	Above FPL	25.2	
		Grandmother	3.9	>HS	17.2			
		Other	2.1	College grad +	9.6	Missing	25.0	

Most parent/caregiver participants who completed the BRFQ were women and most often were mothers of the children in the studies (Table 5). Twenty to 25% of participants completing the BRFQ had less than high school education, and in UCD and UCSF studies only about 10% had some college education or a graduate degree.

The UCD studies took place on tribal reservations and included several items specific to that population. For example, questions were asked about tribal identity and affiliation, and about dental care received through the Indian Health Service. In addition, UCD studies included more variables related to attitudes and psychosocial characteristics, including oral health locus of control [17] sense of coherence, chronic stress, perceived discrimination, distress, and financial stability (Table 3). The investigators felt that it was important to probe the extent to which reservation-dwelling populations, among whom caries rates are extraordinarily high, felt that they had some influence over the oral health of their children. Stress, discrimination, and financial stability measures had been developed and productively used in other studies of American Indian health issues [10, 18], showing that stress is common among American Indian populations living on reservations [18].

The health literacy item used by the BU and UCSF studies (Table 3) was the well-accepted Single Item Literacy Screener [19], which has been cited in over 300 papers. During the measures pilot, UCD study investigators found that in their population the health literacy item was not related to education or income/poverty, constructs that are generally very reliably related to health literacy. Further, the item showed limited variability, with 84% of participants reporting no difficulty with reading medical materials, despite lower levels of educational attainment among American Indian populations. Based on these findings and the fact that the item itself has a relatively high degree of reading difficulty, UCD replaced the Single Item Literacy Screener with slightly adapted versions of three well-validated health literacy screening items [20, 21]. In the UCSF quality management assessment pilot of 40 parent-child dyads, staff found that using a flip chart to display response options improved administration flow in case participants did not remember them. Following the UCSF pilot, the response option of “less than 1 year old” was added to the question about age of child at first dental visit and Spanish translations were refined for clarity. The BU pilot consisted of cognitive interviews of 36 individuals (20 in English and 16 in Spanish) to ensure that the BRFQ was culturally appropriate and at the appropriate literacy level. Similar to the UCSF pilot, the BU study also found that utilizing a flip chart of responses facilitated data collection. To ensure that the final surveys would be as consistent as possible across Centers, decisions about final item refinements were determined collaboratively.

In addition to measuring self-efficacy and perceived importance, BU assessed motivation to engage in a variety of oral health recommendations and reduce ECC (Table 3). The measure was designed to assess the effects of their intervention, which was based on Motivational Interviewing. MI techniques attempt to build motivation by helping participants weigh the pros and cons of change, discussing the discrepancy between their behaviors and values/goals, and helping participants look forward to the positive changes that could result from initiating and sustaining pediatric oral health behaviors.

BU also measured caregiver stress using the Hassles Scale. Previous research has shown that daily hassles may create even more stress than traditional measures of stress focused on major life events [22]. Their predictive value has been shown in low-income populations [23] and in the context of health outcomes [24].

BU and UCSF used the revised multidimensional Acculturation Scale for Mexican-Americans (ARSMA-II) acculturation scale, which measures the degree to which participants are culturally tied to both Anglo and Hispanic communities [25]. Acculturation is correlated with years since immigration and age at immigration. Investigators wanted to be able to include acculturation as a possible effect modifier of the interventions. In addition, UCSF asked about child’s birthplace.

Both BU and UCSF included additional oral health knowledge questions beyond the essential set to ensure that there were knowledge questions that mapped directly to their intervention. For example, BU included questions regarding knowledge about timing of first dental visit and bedtime sippy cup use. UCSF asked additional questions related to its intervention including fluoride exposure, and application of fluoride varnish and dental sealants.

## Discussion

The EC4 was charged with serving as a national resource for information and research on programs and interventions to promote oral health for all children, including the most vulnerable populations. The EC4 is using the BRFQ for studies with large Hispanic, African-American, and American Indian populations. Development of the BRFQ was integral to measuring components of that mission. Creation of this standard set of measures was intended to facilitate comparisons of risk factors and oral health outcomes across disparate populations of young children at risk for ECC.

Despite its value, the BRFQ has limitations related to its primary purpose for use in randomized clinical trials of ECC prevention to identify effective interventions, and not necessarily for identifying risk factors for ECC. A key premise underlying our use of the BRFQ was the expectation that successful randomization would balance the distribution of important baseline risk factors among the different arms of each trial. Thus, the BRFQ does not include variables on a number of important ECC risk predictors such as community water fluoridation. Neither does the BRFQ include detailed assessments of caregiver or child dental fear/anxiety, although these variables may be important determinants of dental service utilization and children’s oral health status. Last but not least, preliminary analyses have suggested between-population differences in the measurement properties of at least some of the BRFQ subscales. The analyses to determine which subscales have invariance across populations are complicated, and it is not possible to summarize those results in this paper, which focuses on the process of developing a set of CDEs in a multi-institution consortium.

To ensure understanding of the full range of determinants and factors contributing to oral health disparities, the BRFQ includes individual-level, parent/family-level, and community-level variables. This approach draws on contemporary knowledge of measures commonly used in oral health and disparities research and includes both identifiable risk factors and structural measures, such as education, income, ethnicity, social structure, and social environments-all of which appear to be major determinants of parental attitudes, beliefs, knowledge and behavior. A number of studies have suggested a strong association of these factors with oral health outcomes [26, 27].

For each study, the relevant university IRB reviewed and approved the BRFQ instrument and protocol used. The BRFQ meets a critical need for standardizing variables of interest across pediatric oral health investigations and facilitating collection of data that can be generalized to the variety of solutions that are sought for addressing disparities in children’s oral health. UCD has published work demonstrating the validity of several BRFQ-based measures in American Indian populations [28–30], and BU has previously published work validating of the POQL [6].

Investigators outside the EC4 have used or adapted the BRFQ in a variety of projects [31], and has been translated into Vietnamese for use with a migrant population in California [32, 33]. The EC4 steering committee has monitored and kept a record of investigators who have used the BRFQ for their projects.

## Conclusion

The process described in this paper may be useful to other multi-institution endeavors such as clinical research networks and consortia developing CDEs [1]. Based on the BRFQ experience, we recommend that future data harmonization or CDE development projects use active working groups with 5–8 engaged members with appropriate training and experience, a chair with good leadership skills, and a set of clear goals, milestones and deadlines. An initial in-person orientation meeting to provide an overview is strongly recommended. Efficient conference call meetings every 2 weeks with agendas and notes from previous calls facilitate working group progress. Finally, pilot testing the resulting CDEs is a critically important step.

As knowledge about ECC continues to advance, additional items may need to be added to the BRFQ, and the inclusion of some current items may be reconsidered. Measures of diet/sugar consumption, fluoride exposure, and additional community-level influences may be warranted, for example. The BRFQ does not include certain key information that could be obtained from a clinical examination of the child or parent/caregiver or from biologic or microbiome samples or genomic or metabolomic analyses.

Additional uses of the BRFQ will contribute to the evidence base needed to determine the most effective ways to prevent ECC. It will be a good practice for any study adopting any published scale to also test the scale in its own population to confirm psychometrics. Finally, adaptations may be needed to reflect oral health related behaviors and attitudes within other cultures or for factors associated with caries incidence in older age groups.

## Abbreviations

BRFQ: Basic Research Factors Questionnaire
BU: Boston University
CDEs: Common Data Elements
DCC: Data Coordinating Center
EC4: Early Childhood Caries Collaborating Centers
ECC: Early Childhood Caries
FPL: Federal Poverty Level
HS/GED: High school/general educational development
NHANES: National Health and Nutrition Examination Survey
NHIS: National Health Interview Survey
NIDCR: National Institute of Dental and Craniofacial Research
POQL: Pediatric Oral Health Related Quality of Life
UCD: University of Colorado Denver
UCSF: University of California, San Francisco
